# Volume change pattern of decompression of mandibular odontogenic keratocyst

**DOI:** 10.1186/s40902-018-0184-y

**Published:** 2019-01-07

**Authors:** Jin Hoo Park, Eun-Jung Kwak, Ki Sung You, Young-Soo Jung, Hwi-Dong Jung

**Affiliations:** 1 Department of Oral and Maxillofacial Surgery, College of Dentistry, Yonsei University, 50-1 Yonsei-ro, Seodaemun-gu, Seoul, 120-752 Korea; 2 Oral Science Research Institute, College of Dentistry, Yonsei University, Seoul, Korea; 3 Department of Applied and Computational Mathematics and Statistics, University of Notre Dame, Indiana, USA

**Keywords:** Odontogenic keratocyst, Keratocystic odontogenic tumor, Decompression, Enucleation

## Abstract

**Objectives:**

This study was aimed to analyze the reducing pattern of decompression on mandibular odontogenic keratocyst and to determine the proper time for secondary enucleation.

**Materials and methods:**

Seventeen patients with OKC of the mandible were treated by decompression. Forty-five series of CT data were taken during decompression and measured by using InVivo software (Anatomage, San Jose, Calif) and were analyzed.

**Results:**

The expected relative volume during decompression is calculated using the following formula: *V*(*t*) = *V*_initial_ × exp.(*at* + 1/2*bt*^2^) (*t* = duration after decompression (day)). There was no significant directional indicator in the rate of reduction between buccolingual and mesiodistal widths.

**Conclusion:**

The volume reduction rate gradually decreased, and 270 days were required for 50% volume reduction following decompression of OKC. The surgeon should be aware of this pattern to determine the timing for definitive enucleation.

**Clinical relevance:**

The volume reduction rate and pattern of decompression of the OKC can be predicted and clinicians should be considered when treating OKC via decompression.

## Background

An odontogenic kearatocyst (OKC) is a rare and benign but locally aggressive developmental cyst [1] and is also called as keratocystic odontogenic tumor. Under the microscope, OKCs vaguely resemble keratinized squamous epithelium. However, they lack rete ridges and often have an artefactual separation from their basement membrane. Treatment of OKC has stirred controversy because of the tumor’s aggressive behavior and high recurrence rate.

The two common treatments of OKC at present are enucleation and decompression [2]. Enucleation followed by application of Carnoy’s solution, liquid nitrogen cryotherapy, and radical surgery with resection is one of treatment of OKC [3]. However, these treatment methods may entail several complications. Alternative treatments are decompression which creates a cystic cavity which connects with the oral cavity [4] and is able cystic fluid to come out. After some decompression period, surgical enucleation is followed by decompression. If the lesion enlarges and approaches a vital structure, decompression is more recommended [5, 6].

This study was aimed to analyze the reducing pattern of decompression on mandibular OKC in terms of direction and speed of volume reduction and to determine the proper timing for secondary enucleation.

## Materials and methods

This study conforms to the Declaration of Helsinki on medical protocol and ethics and was approved by the regional Ethical Review Board of Yonsei University Dental Hospital Institutional Review Board (IRB 2-2014-0031).

There were 90 subjects who were diagnosed with OKC from 2007 to 2013 at the Department of Oral and Maxillofacial Surgery, Yonsei University Dental Hospital. The exclusion criteria were other cystic lesions such as radicular cyst, dentigerous cyst, and unicystic ameloblastoma; an existing medical, physical, or mental condition that would impair the healing potential; syndromic craniofacial lesion such as basal cell nevus syndrome; or the absence of any of digitized pre-/post-operative CT and the site of lesion was limited only within mandible. Seventeen patients were included for this study base on the above inclusion and exclusion criteria.

**Fig. 1:**
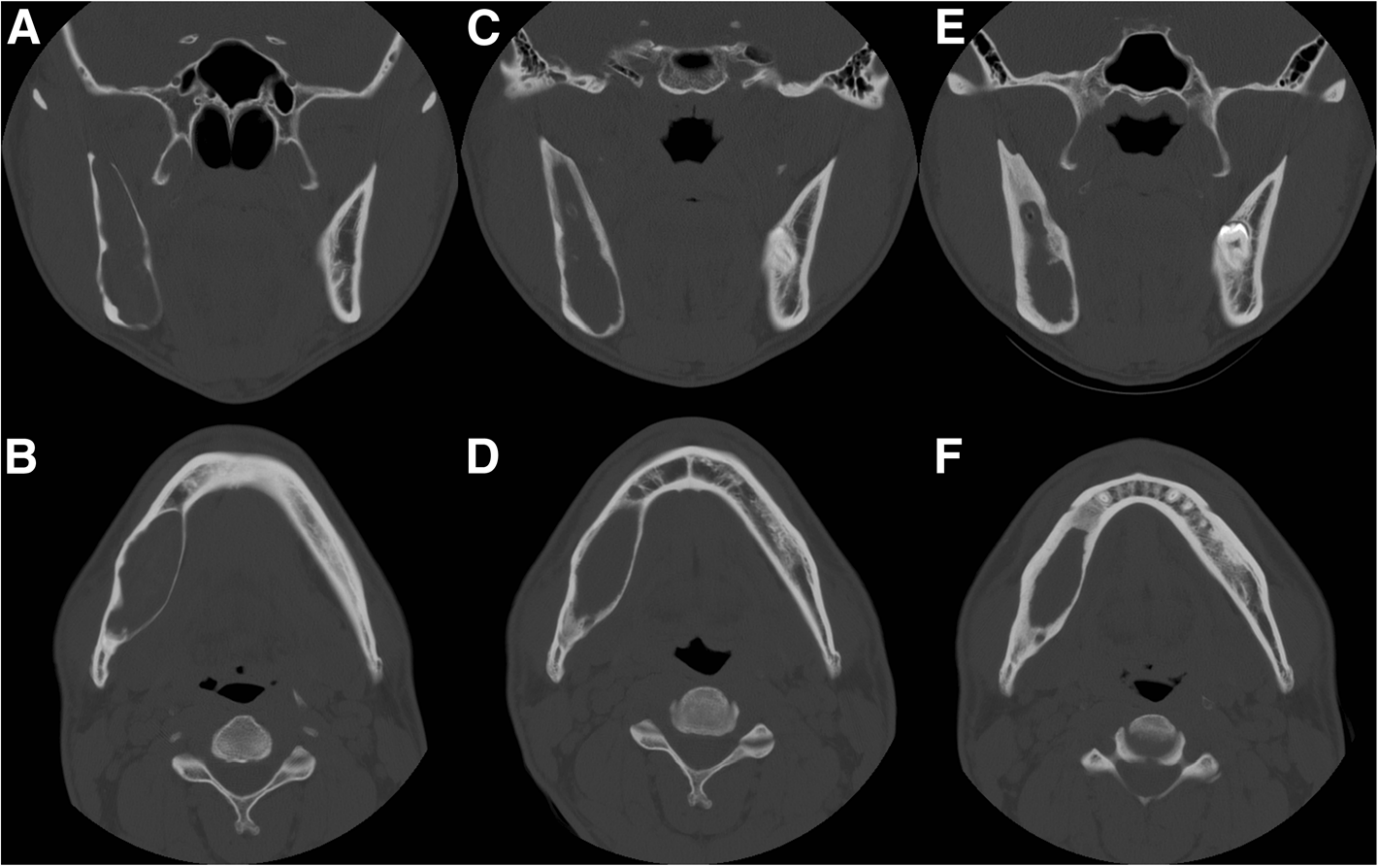
Representive serial axial and coronal CT of OKC decompression. During the decompression for about 18 months, remarkable reduction in lesion size and increased cortical bone thickness with new bone formation is observed. (**a**, **b** initial, **c**, **d** 6 months decompression, **e**, **f** 18 months decompression)

Patients preliminarily diagnosed with OKC underwent an incisional biopsy of the cyst at the date of decompression. The patient’s individual appliance for decompression tube was fabricated and located to the punctured area. During the follow-up period, self-irrigation through the decompression tube was applied. Surgeons checked reduction of cystic volume and bone formation via clinical and radiographic examination (panoramic view) every 2–3 months. When the cystic fluid exudate lessened clinically and radiography indicated the tumor had shrunk from important anatomical structures such as the inferior alveolar nerve, surgeons stopped decompression and began planning to enucleate the OKC (Fig. 1).

**Fig. 2:**
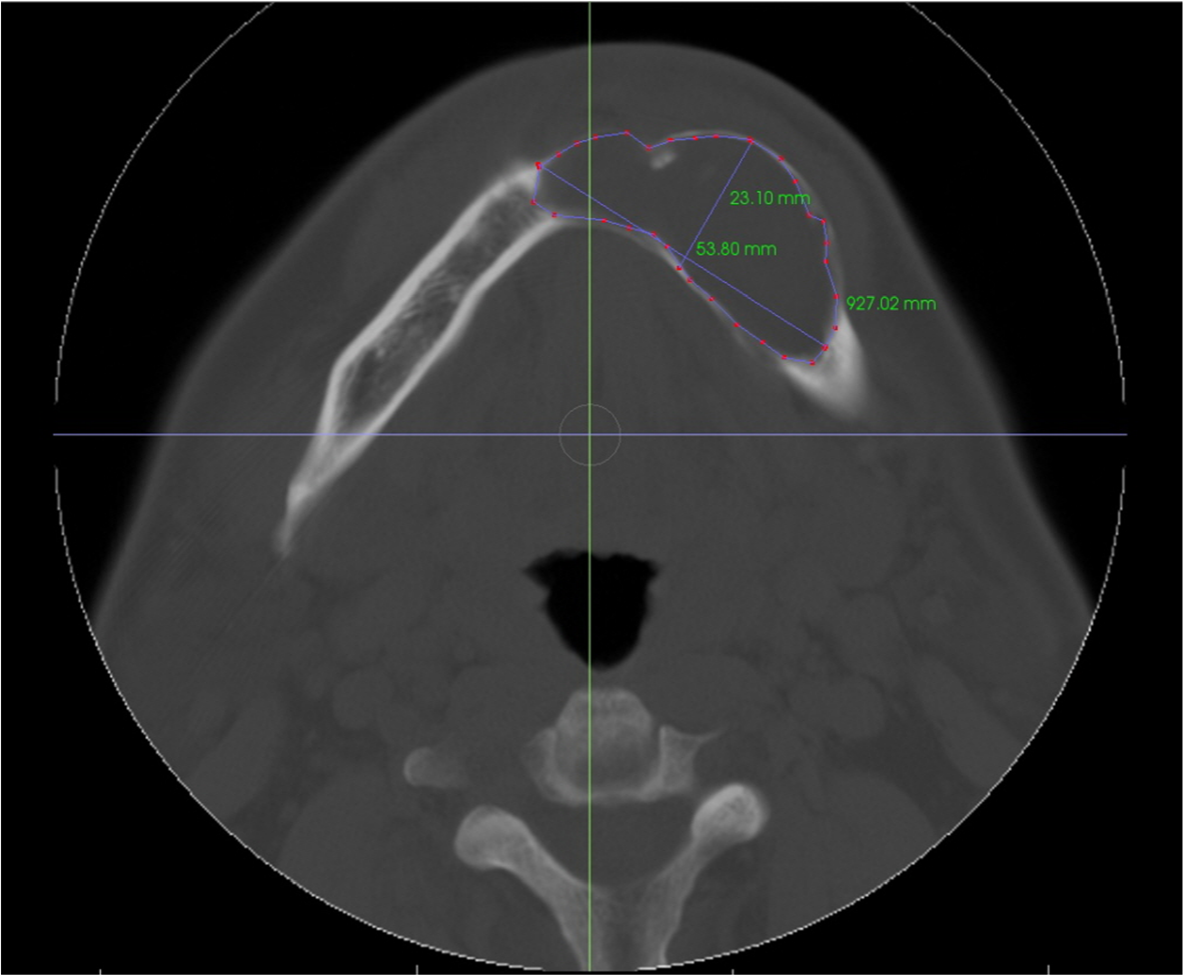
The size of an area in axial cut and BL, MD width of lesion is measured

Measurement data such as volume and other metrics was collected by InVivo software (Anatomage, San Jose, Calif). Due to the intrinsic difficulty of applying automated image processing algorithms, heuristic approaches were widely applied in the data acquisition process. The size of the area was measured from indicated reference points along the cystic lesion in every axial cut of CT images. Volume was then approximated using Riemannian integration by summing up area values from each layer. The buccolingual (BL) and mesiodistal (MD) widths were measured in a similar manner from an image with the maximum area (Fig. 2). The three different investigators independently collected the data and analyzed it with the average values. The authors evaluated the effect of decompression of OKC in terms of volume change as well as MD and BL widths.

**Fig. 3:**
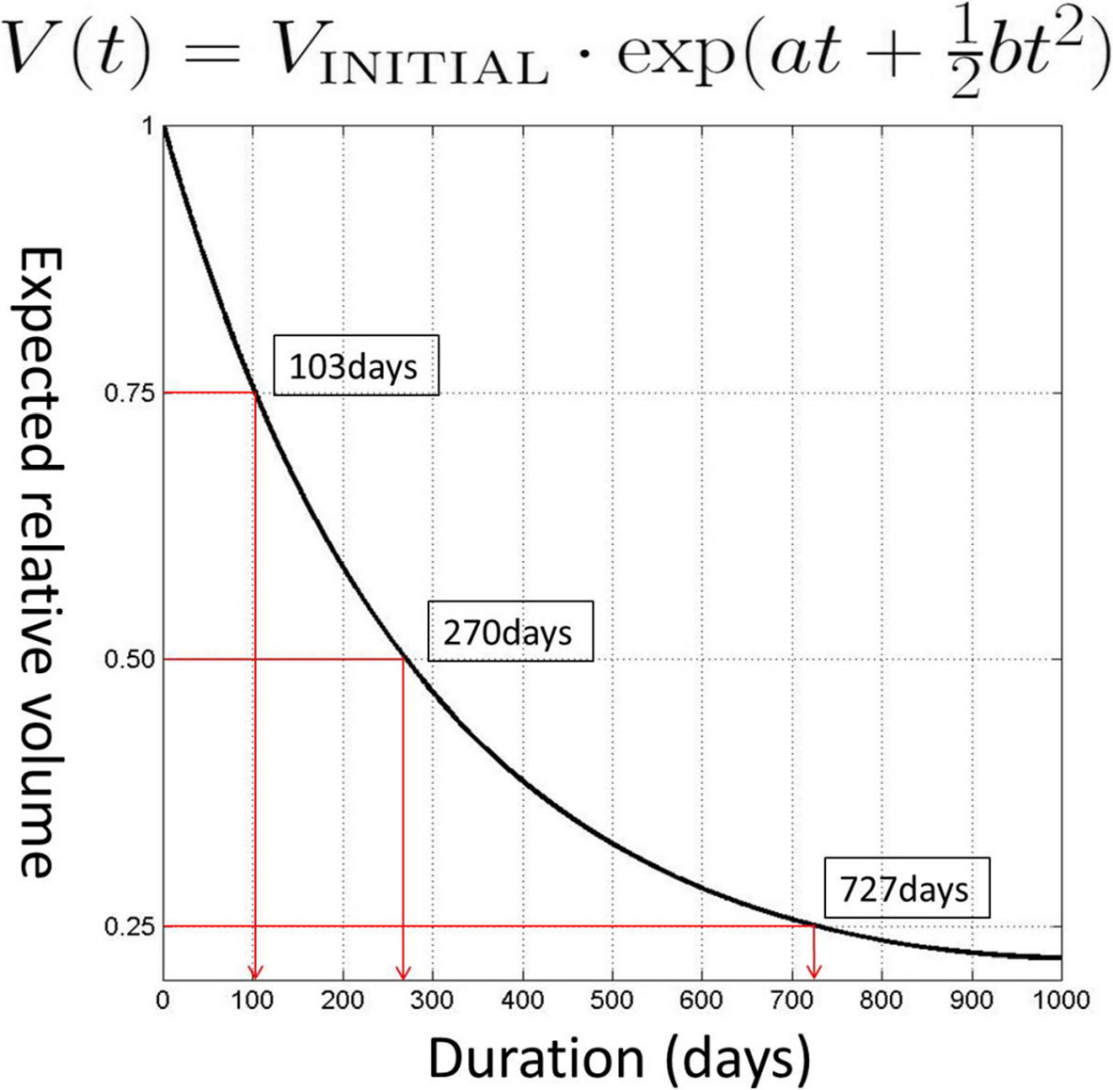
The expected relative volume

Estimation of coefficients *ɑ* and *b* in the model was performed by standard regression in terms of square error (Fig. 3). Intrinsic assumption of normality of data was tested using Shapiro-Wilks test and other posterior verification methods, including QQ plot and residual test.

## Results

The study group consisted of 17 patients, 8 males and 9 females, of average age 33.7 years (range 16–79 years). The average age of the 8 males was 29.8 years, and 9 females was 37.2 years. OKC prevalence was highest in the second and third decade (9/17 patients). The average initial volume was 34,020.85mm^3^ and after decompression volume was 14,007.88 mm^3^. The average treatment period for all patients is 298 days of decompression (Table 1). The following formula presents the prediction of volume reduction according to elapsed time (Fig. 3). The volume of OKC undergoing decompression was reduced by 25, 50, and 75% over 103, 270, and 727 days (Fig. 3).

**Table 1:** Demographic of patients’ information

Male: female	1: 1.12	
Age	33.72	± 18.00
Initial volume	34,020.85 mm^3^	± 20,778.57
Volume reduction rate	56.05%	± 16.83
Duration of decompression	298.35 days	± 183.02

**Fig. 4:**
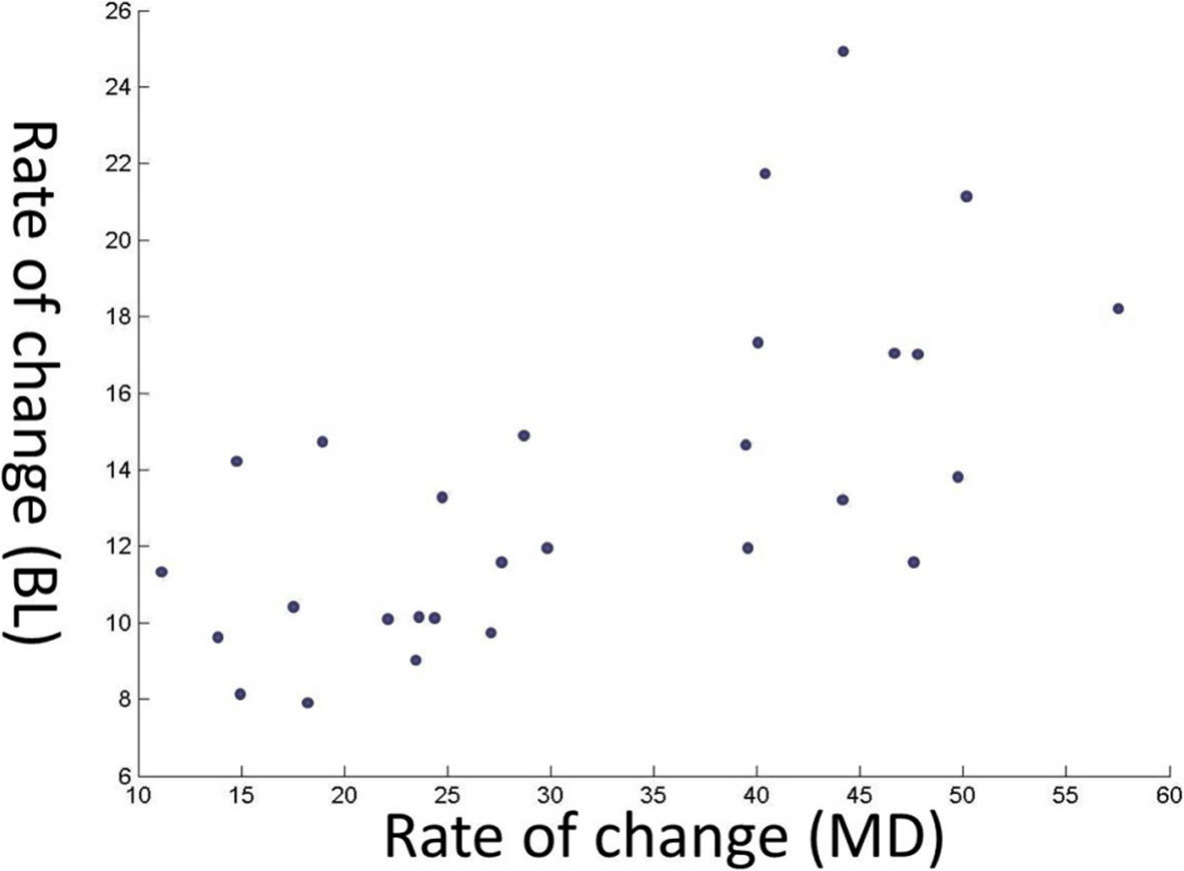
Relation between BL and MD widths

**Fig. 5:**
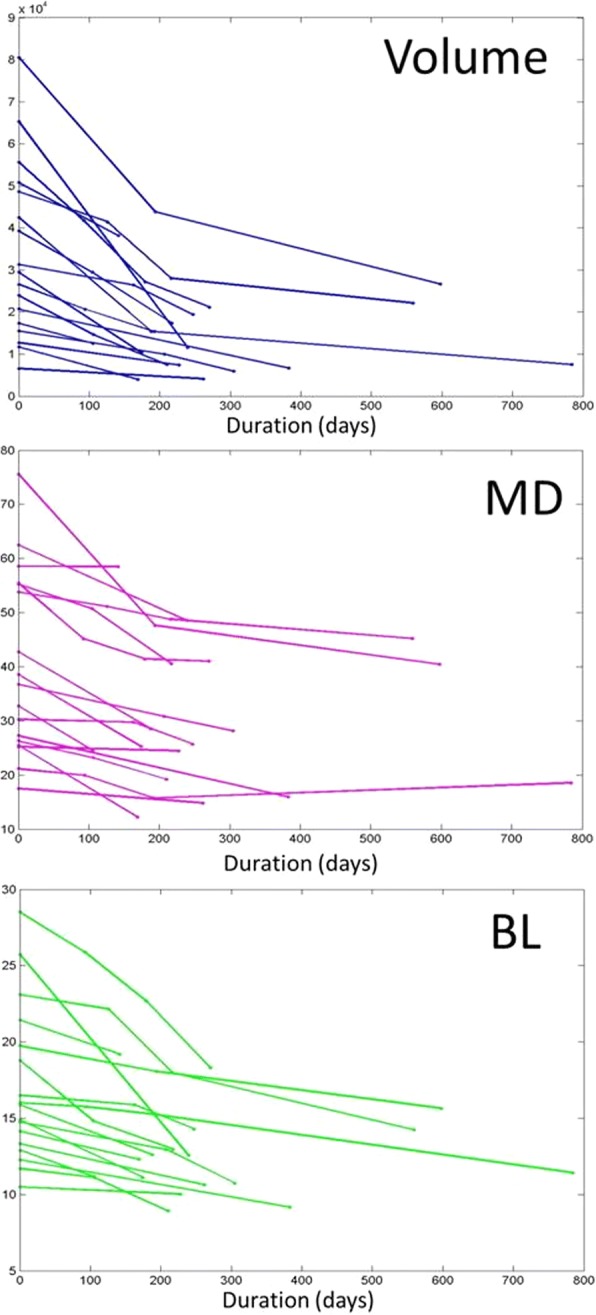
The changes of volume, BL, and MD diameter

There was no significant directional indicator in the rate of reduction between buccolingual and mesiodistal widths (correlation coefficient 0.6626) (Fig. 4). The changes in volume as well as MD and BL diameters during the decompression period are shown (Fig. 5).

The average follow-up period is 776 days (2.1 years). Among 17 patients, the recurrence case is only 1 patient, observed 6.5 years after surgery.

## Discussion

OKC is one of the most aggressive cyst among odontogenic cystic lesions. Though OKC is not known to metastasize, it can induce resorption, expansion of the jaw and change of the facial profile as it grows. Although in comparison with a small number of studies, prevalence in this study was higher in 10 to 30-year-old patients, consistent with a previous study [7]. One-third of patients were asymptomatic and usually diagnosed by routine dental examination; 50% of them complained of jaw swelling and mild pain [8, 9]. Though rare, conversion to malignant deviation such as primary intraosseous squamous cell carcinoma (PIOSCC) of the jaw is possible.

There are several studies on the differences in recurrence according to the treatment modalities of OKC. Kaczmarzyk et al. report that, the overall recurrence rate of OKC was 23.15%. The recurrence rates were followed by treatment modalities were 0% for resection, 0% for enucleation with peripheral ostectomy and Carnoy’s solution, 18.18% for enucleation with peripheral ostectomy, 26.09% for enucleation alone, 40% for marsupialization without following enucleation, and 50% for enucleation with Carnoy’s solution [10]. The most obvious way to prevent recurrence of OKC is resection. But this is a very invasive surgical procedure. In a review study of OKC in Blanas et al., simple enucleation was reported to have a recurrence rate of 17% to 56% [11]. The recurrence rate of simple enucleation of OKC is very high. However there are several reports of a low recurrence rate after decompression following enucleation of OKC (0–8.7%) [4, 12–14].

The decompression and second-stage enucleation of OKC might be the first choice of treatment in terms of functional and esthetic reconstruction. In cases of huge cystic size and proximity with vital anatomic structures, decompression is the most proper treatment. During the decompression period, an opening by appliance allows for drainage of fluid which consists of OKC. This leads to decreased intracystic pressure [15] and bone formation along the periphery of the cystic wall, regardless of the direction of either MD or BL. Decompression and second-stage enucleation can thus be deemed beneficial in terms of decreased complications (nerve injury, pathologic fracture, discomfort).

The volume of OKC was reduced by 25, 50, and 75% over 103, 270, and 727 days of decompression. The surgeons in our department decided timing for enucleation based on the amount of discharging fluid and detachment from important anatomical structure in panoramic and CT images. The average decompression duration of study group was 298 days in this way. The time to enucleation based on the reduced exudate clinically and volume change as observed by the surgeon (298 days) was similar with the time of 50% reduction rate of OKC (270 days). Therefore, enucleation time set by the surgeon seems appropriate.

Tube length during decompression should be continuously adjusted as the OKC decreases in size. Amount of decrease can be confirmed by both panoramic view and CT. However, investigation by panoramic view is limited as evaluation of buccolingual dimension and volume are close to impossible [15]. In this respect, CT yields more information than 2D imagery. Therefore, panoramic images should be taken during periodic follow-up, CT being taken when there is a necessity for more accurate confirmation.

A relatively low recurrence rate was observed in this study when decompression was followed by enucleation. Moreover, the final pathology report was coincided with that of incisional biopsy performed in decompression procedure.

## Conclusion

As a treatment of OKC, decompression followed by enucleation was more conservative and effective than other procedures. Decompression preserves the important anatomical structures of the jaw and thus decreases complications such as functional and esthetic damage [16]. This study provides novelty in that it can predict the volume reduction of the decompressed OKC, which reduces in a half-life of 270 days. Although the rate of recurrence is low using this procedure, periodic check-ups are necessary.

## Abbreviations

BL: Buccolingual
CT: Computed tomography
KCOT: Keratocystic odontogenic tumor
MD: Mesiodistal
OKC: Odontogenic keratocyst
PIOSCC: Primary intraosseous squamous cell carcinoma
